# Risk Factors for and Health Status of Socially Isolated Adults

**DOI:** 10.1001/jamanetworkopen.2024.57330

**Published:** 2025-01-30

**Authors:** Tarun Ramesh, Kushal Kadakia, Marcela Horvitz-Lennon, Joshua Breslau, Hao Yu

**Affiliations:** 1 Harvard Medical School, Boston, Massachusetts; 2 Department of Population Medicine, Harvard Medical School and Harvard Pilgrim Health Care Institute Landmark Center, Boston, Massachusetts; 3 RAND Corporation, Boston, Massachusetts

## Abstract

This cross-sectional study examines the risk factors associated with social isolation among US adults and the health status of those who report being socially isolated using national surveillance data.

## Introduction

In 2023, the Surgeon General issued an advisory on the epidemic of loneliness and social isolation, calling for more research and policy interventions to address these challenges.^1,2^ Prior studies have identified that social isolation increases cardiovascular risk and stroke mortality.^3,4^ Researchers have used surveillance data to examine social isolation among sexual and gender minority groups.^5^ However, there has been limited research using national surveillance data to broadly examine risk factors for social isolation and self-reported health status among socially isolated adults in the US.

## Methods

This cross-sectional study was exempted by the Harvard Pilgrim Health Care Institute institutional review board because we used publicly available, deidentified data. We followed the Strengthening the Reporting of Observational Studies in Epidemiology (STROBE) reporting guideline.

This study analyzed secondary data from the 2022 Behavioral Risk Factor Surveillance System (BRFSS). We defined socially isolated adults as those who responded always when asked “How often do you feel socially isolated from others?” The survey also asked questions about the numbers of poor physical health days and poor mental health days in the past 30 days (eMethods in Supplement 1).

We first conducted a logistic regression on whether an adult always felt socially isolated using sociodemographic and chronic health condition diagnoses as covariates. Then, we specified 2 negative binomial models to estimate numbers of poor physical health and poor mental health days in the past 30 days, comparing socially isolated to not socially isolated adults after controlling for sociodemographic and chronic health conditions (eMethods in Supplement 1). All models accounted for the BRFSS sampling design variables of weight, strata, and sampling unit.^6^ Statistical analysis was conducted in Stata version 18.0 (StataCorp) and analyzed from July to August 2024. Statistical significance was set at *P* < .05, and all tests were 2-sided.

## Results

In our sample of 251 125 adults (133 927 female [53.3%]; 26 187 Hispanic adults [10.4%]; 193 186 non-Hispanic White adults [76.9%]; 69 758 aged 35 to 54 years [27.8%]), 8098 adults (3.22%) felt socially isolated (Table). Non-Hispanic Black adults (adjusted odds ratio [aOR], 1.70; 95% CI, 1.43-2.03), non-Hispanic American Indian or Alaska Native adults (aOR, 1.78; 95% CI, 1.12-2.84), and Hispanic adults (aOR, 1.34; 95% CI, 1.11-1.61) had a greater likelihood of social isolation compared with non-Hispanic White adults. Other risk factors included living in a nonmetropolitan county (aOR, 1.19; 95% CI, 1.02-1.38), being single (aOR, 1.21; 95% CI, 1.05-1.40), living alone (aOR, 1.21; 95% CI, 1.04-1.40), being unemployed for 1 year or more (aOR, 1.98; 95% CI, 1.44-2.74), being uninsured (aOR, 1.57; 95% CI, 1.25-1.98), and having low personal income (less than $25 000) (Table).

**Table.** Characteristics of Adults Who Were and Were Not Socially Isolated^a^

Characteristic	Adults, No. (%)		*P* value	Standardized mean difference	aOR (95% CI)	*P* value
	Not socially isolated (n = 243 027 [96.8%])	Socially isolated (n = 8098 [3.2%])				
Sex						
Female	113 251 (46.6)	3947 (48.7)	<.001	0.043	0.80 (0.70-0.90)	<.001
Male	129 776 (53.4)	4151 (51.3)			1 [Reference]	NA
Race and ethnicity^b^						
Hispanic	24 767 (10.2)	1420 (17.5)	<.001	0.341	1.34 (1.11-1.61)	.002
Non-Hispanic						
American Indian or Alaska Native	3296 (1.4)	259 (3.2)			1.78 (1.12-2.84)	.01
Asian	5155 (2.1)	141 (1.7)			1.38 (0.98-1.93)	.06
Black	17 024 (7.0)	898 (11.1)			1.70 (1.43-2.03)	<.001
Native Hawaiian or Pacific Islander	457 (0.2)	23 (0.3)			1.04 (0.47-2.30)	.92
White	188 094 (77.4)	5092 (62.9)			1 [Reference]	NA
Multiracial	4234 (1.7)	265 (3.3)			0.99 (0.74-1.31)	.92
Age categories, y						
18-35	37 606 (15.5)	1529 (18.9)	<.001	0.217	1 [Reference]	NA
35-54	67 109 (27.6)	2649 (32.7)			0.87 (0.73-1.03)	.10
55-64	45 775 (18.8)	1644 (20.3)			0.67 (0.54-0.83)	<.001
≥65	92 537 (38.1)	2276 (28.1)			0.58 (0.44-0.77)	<.001
Education						
Did not graduate high school	12 869 (5.3)	1237 (15.4)	<.001	0.539	1 [Reference]	NA
Graduated high school	56 650 (23.4)	2602 (32.3)			0.75 (0.62-0.90)	.002
Attended college or technical school	66 731 (27.6)	2431 (30.2)			0.62 (0.51-0.76)	<.001
Graduated from college or technical school	105 860 (43.7)	1780 (22.1)			0.43 (0.35-0.54)	<.001
Residence						
Nonmetropolitan	66 841 (28.2)	2396 (30.9)			1.19 (1.02-1.38)	.03
Metropolitan	169 786 (71.8)	5357 (69.1)	<.001	0.058	1 [Reference]	NA
Income, $						
<25 000	29 797 (15.0)	2710 (41.9)	<.001	0.781	1 [Reference]	NA
25 000-35 000	23 166 (11.6)	1083 (16.7)			0.85 (0.70-1.02)	.08
35 000-50 000	26 561 (13.4)	868 (13.4)			0.76 (0.63-0.93)	.007
50 000-100 000	62 720 (31.5)	1171 (18.1)			0.54 (0.43-0.67)	<.001
100 000-200 000	43 013 (21.6)	493 (7.6)			0.41 (0.31-0.55)	<.001
>200 000	13 695 (6.9)	141 (2.2)			0.42 (0.27-0.64)	<.001
Marriage status						
Single	102 411 (42.5)	5144 (64.3)	<.001	0.449	1.21 (1.05-1.40)	.01
Married or partnered	138 682 (57.5)	2854 (35.7)			1 [Reference]	NA
Lives alone	61 503 (25.5)	2820 (35.1)	<.001	0.212	1.21 (1.04-1.40)	.01
Employment status						
Employed	102 221 (42.4)	2335 (29.2)	<.001	0.645	1 [Reference]	NA
Self-employed	21 463 (8.9)	609 (7.6)			1.07 (0.85-1.36)	.54
Out-of-work for 1 y or more	4348 (1.8)	413 (5.2)			1.98 (1.44-2.74)	<.001
Out-of-work for less than 1 y	4154 (1.7)	294 (3.7)			1.11 (0.83-1.50)	.48
Homemaker	9823 (4.1)	397 (5.0)			0.92 (0.69-1.24)	.58
Student	5583 (2.3)	182 (2.3)			0.85 (0.56-1.30)	.46
Retired	79 983 (33.2)	1805 (22.6)			0.85 (0.67-1.08)	.18
Unable to work	13 357 (5.5)	1951 (24.4)			1.94 (1.55-2.41)	<.001
Insurance coverage						
Private insurance	109 006 (46.5)	2036 (26.4)	<.001	0.544	1 [Reference]	NA
Medicaid	14 191 (6.1)	1251 (16.2)			1.30 (1.01-1.66)	.04
Medicare	77 908 (33.2)	2443 (31.7)			1.04 (0.83-1.30)	.74
Other insurance	21 634 (9.2)	1089 (14.1)			1.20 (0.98-1.46)	.08
Uninsured	11 720 (5.0)	883 (11.5)			1.57 (1.25-1.98)	<.001
Chronic condition diagnosis						
Cancer	28 914 (12.0)	941 (11.8)	0.6	0.006	1.28 (1.04-1.59)	.02
COPD	18 860 (7.8)	1505 (18.8)	<.001	0.328	1.49 (1.27-1.74)	<.001
Chronic kidney disease	11 047 (4.6)	596 (7.4)	<.001	0.121	1.35 (1.07-1.71)	.01
Myocardial infarction or congestive heart failure	21 664 (9.0)	1192 (15.1)	<.001	0.188	1.11 (0.92-1.33)	.27
Stroke	10 139 (4.2)	700 (8.7)	<.001	0.185	0.94 (0.76-1.17)	.60
Depression	49 443 (20.5)	3878 (48.6)	<.001	0.619	2.69 (2.37-3.06)	<.001

Abbreviations: COPD, chronic obstructive pulmonary disease; NA, not available.

^a^ We defined socially isolated individuals as those who responded “always” when asked “How often do you feel socially isolated from others?”

^b^ Race and ethnicity were self-reported in the Behavioral Risk Factor Surveillance System survey and used as sociodemographic information.

Chronic condition diagnoses, including cancer (aOR, 1.28; 95% CI, 1.04-1.59), chronic obstructive pulmonary disease (aOR, 1.49; 95% CI, 1.27-1.74), chronic kidney disease (aOR, 1.35; 95% CI, 1.07-1.71), and depression (aOR, 2.69; 95% CI, 2.37-3.06), increased the likelihood of social isolation (Table). Socially isolated adults had significantly more mean days of poor physical health (6.00 [95% CI, 5.44- 6.57] days vs 4.28 [95% CI, 4.18-4.38] days; *P* < .001; standardized mean difference [SMD], 0.019) and poor mental health (8.61 [95% CI, 7.94-9.27] days vs 4.79 [95% CI, 4.70-4.88] days; *P* < .001; SMD, 0.036) in the past 30 days compared with non–socially isolated adults, adjusted for covariates (Figure).

**Figure.**
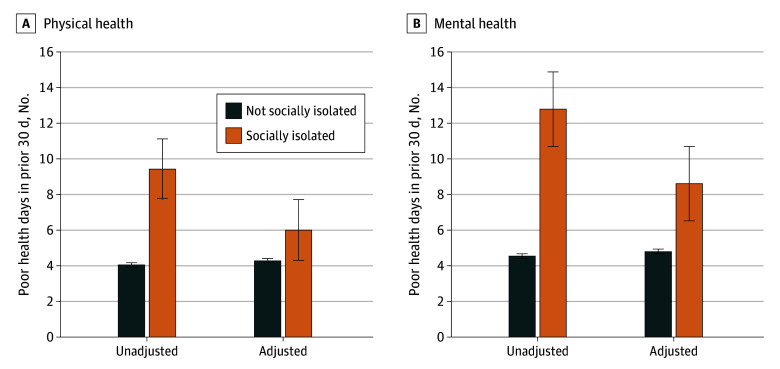
Poor Physical and Mental Health Days in Prior 30 Days The adjusted number of days included the following covariates: female (reference: male), race (reference: non-Hispanic White), age categories (reference: ages 18 to 35 years), educational attainment level, nonmetropolitan county, income, marital status, lives alone, employment status, health insurance coverage, cancer, chronic obstructive pulmonary disease, chronic kidney disease, myocardial infarction or congestive heart failure, stroke, and depression. Whiskers indicate standard errors.

## Discussion

The overall prevalence of social isolation in our study was 3%, which is lower than other determinants of health, such as smoking, poverty, and inadequate health insurance. Our results indicate 3 broad and likely interrelated populations at risk for social isolation, including racial and ethnic minority groups, those with financial insecurity (ie, unemployed, uninsured, lower income), and those with chronic health conditions, with depression being a large factor. We also found that the socially isolated adults reported worse health status compared with those without social isolation. However, the directionality of the association between social isolation and health status is unclear, since adults who are feeling mentally or physically unwell may feel more socially isolated.

Limitations to our study include having only 1 year of data, being cross-sectional, focusing on adults, and relying on self-reported health status. Further research should track trends in social isolation among both adults and children; policy interventions should prioritize populations at risk for social isolation.

## Notes

Supplement 1.
eMethods.

eReferences.

Supplement 2.
Data Sharing Statement
