# Supplementary material for: Risk Factors for and Health Status of Socially Isolated Adults
Source: JAMA Netw Open. 2025 Jan 30;8(1):e2457330. doi: 10.1001/jamanetworkopen.2024.57330 (PMC11783191; doi:10.1001/jamanetworkopen.2024.57330)
Supplement: Supplement 1. — eMethods. eReferences. [file jamanetwopen-e2457330-s001.pdf]

Supplemental Online Content

Ramesh T, Kadakia K, Horvitz-Lennon M, Breslau J, Yu H. Risk factors for and health status of socially isolated adults. *JAMA Netw Open*. 2025;8(1):e2457330. doi:10.1001/jamanetworkopen.2024.57330

eMethods
eReferences

This supplemental material has been provided by the authors to give readers additional information about their work.

© 2025 Ramesh T et al. *JAMA Network Open*.

eMethods.
The Behavior Risk Factor Surveillance system (BRFSS) collects information about chronic health conditions and risk factors for US residents using a telephone household survey for adults 18 or older. In 2022, the survey had a 45% response rate. Multiple studies have previously used BRFSS including to identify e-cigarette use,[1] transgender health,[2] and lung cancer screenings.[3] The 2022 BRFSS included a question in the social determinants and health equity module asking, "How often do you feel socially isolated from others?" We defined socially isolated individuals as respondents who answered "Always" in the survey. 2022 was the first year that the question on social isolation was asked. The following states were administered the question including: Alabama, Alaska, Arizona, California, Connecticut, Delaware, District of Columbia, Florida, Georgia, Idaho, Indiana, Iowa, Kansas, Kentucky, Maine, Maryland, Massachusetts, Michigan, Minnesota, Mississippi, Missouri, Montana, Nebraska, Nevada, New Hampshire, New Jersey, New Mexico, North Carolina, Ohio, Oklahoma, Puerto Rico, Rhode Island, South Carolina, Tennessee, Texas, Utah, Vermont, Virgin Islands, Washington, West Virginia, Wisconsin, and Wyoming.[4]

Living alone was determined as a dichotomous variable, which included three questions: "How many children less than 18 years of age live in your household?" Excluding adults living away from home, such as students away at college, how many members of your household, including yourself, are 18 years of age or older?" for landline surveys, and "How many members of your household, including yourself, are 18 years of age or older?" for cell phone surveys. Respondents had to answer that there were no other adults or children in the household to be considered living alone.[5]

Covariates for both logistic regression and adjusted negative binominal models included the following: sex, race and ethnicity, age categories, education level, metropolitan status, income categories, marital status, employment, living alone, uninsured status, cancer, chronic obstructive pulmonary disease, chronic kidney disease, myocardial infarction/ congestive heart failure, and depression.

The number of poor physical, mental, and both physical and mental health days were ascertained by the following questions:[6]

1. "Now thinking about your physical health, which includes physical illness and injury, for how many days during the past 30 days was your physical health not good?"
2. "Now thinking about your mental health, which includes stress, depression, and problems with emotions, for how many days during the past 30 days was your mental health not good?"
3. "During the past 30 days, for about how many days did poor physical or mental health keep you from doing your usual activities, such as self-care, work, or recreation?"

Like a prior study using BRFSS to study social isolation, participants who responded, "don't know/not sure," refused to answer, or had missing responses for demographic or health variables were excluded.[7]

© 2025 Ramesh T et al. *JAMA Network Open*.

eReferences.

1. Erhabor J, Boakye E, Obisesan O, et al. E-Cigarette Use Among US Adults in the 2021 Behavioral Risk Factor Surveillance System Survey. *JAMA Netw Open.* 2023;6(11):e2340859. doi:10.1001/jamanetworkopen.2023.40859
2. Baker KE. Findings From the Behavioral Risk Factor Surveillance System on Health-Related Quality of Life Among US Transgender Adults, 2014-2017. *JAMA Intern Med.* 2019;179(8):1141–1144. doi:10.1001/jamainternmed.2018.7931
3. Henderson LM, Su I, Rivera MP, et al. Prevalence of Lung Cancer Screening in the US, 2022. *JAMA Netw Open.* 2024;7(3):e243190. doi:10.1001/jamanetworkopen.2024.3190
4. CDC – BRFSS – 2022 BRFSS Modules Used by Category. Centers for Disease Control and Prevention. Available at: https://www.cdc.gov/brfss/questionnaires/modules/category2022.htm
5. Das Gupta D, Kelekar U, Rice D. Associations between living alone, depression, and falls among community-dwelling older adults in the US. Prev Med Rep. 2020 Dec 2;20:101273. doi: 10.1016/j.pmedr.2020.101273. PMID: 33354494; PMCID: PMC7744925.
6. 2022 BRFSS Survey Data and Documentation. Centers for Disease Control and Prevention. Available at: https://www.cdc.gov/brfss/annual_data/annual_2022.html
7. Bruss KV, Seth P, Zhao G. Loneliness, Lack of Social and Emotional Support, and Mental Health Issues — United States, 2022. MMWR Morb Mortal Wkly Rep 2024;73:539–545.

© 2025 Ramesh T et al. *JAMA Network Open*.
