# Supplementary material for: Risk Factors for and Health Status of Socially Isolated Adults
Source: JAMA Netw Open. 2025 Jan 30;8(1):e2457330. doi: 10.1001/jamanetworkopen.2024.57330 (PMC11783191; doi:10.1001/jamanetworkopen.2024.57330)
Supplement: Supplement 2. — Data Sharing Statement [file jamanetwopen-e2457330-s002.pdf]

Data Sharing Statement

Data
Data available: Yes
Data types: Data (not involving human participants)
How to access data: The Behavioral Risk Factor Surveillance System (BRFSS) data are publicly available at: https://www.cdc.gov/brfss/data_documentation/index.htm
When available: With publication

Supporting Documents
Document types: None

Additional Information
Who can access the data: Anyone requesting the data
Types of analyses: For any purpose
Mechanisms of data availability: Without investigator support
